# Short and Long Time Bloodstains Age Determination by Colorimetric Analysis: A Pilot Study

**DOI:** 10.3390/molecules26206272

**Published:** 2021-10-16

**Authors:** Alessandro Marrone, Daniele La Russa, Alberto Montesanto, Vincenzo Lagani, Mauro F. La Russa, Daniela Pellegrino

**Affiliations:** 1 Department of Biology, Ecology and Earth Sciences, University of Calabria, 87036 Rende, Italy; alberto.montesanto@unical.it (A.M.); mauro.larussa@unical.it (M.F.L.R.); danielapellegrino@unical.it (D.P.); 2 Department of Pharmacy, Health and Nutritional Sciences, University of Calabria, 87036 Rende, Italy; daniele.larussa@unical.it; 3 Institute of Chemical Biology, Ilia State University, 0162 Tbilisi, Georgia; vincenzo.lagani@iliauni.edu.ge

**Keywords:** bloodstains, bloodstain age estimation, colorimetric analysis

## Abstract

Bloodstains found at crime scenes represent a crucial source of information for investigative purposes. However, in forensic practice, no technique is currently used to estimate the time from deposition of bloodstains. This preliminary study focuses on the age estimation of bloodstains by exploiting the color variations over time due to the oxidation of the blood. For this purpose, we used a colorimetric methodology in order to easily obtain objective, univocal and reproducible results. We developed two bloodstain age prediction algorithms: a short-term and a long-term useful model for the first 24h and 60 days, respectively. Both models showed high levels of classification accuracy, particularly for the long-term model. Although a small-scale study, these results improve the potential application of colorimetric analysis in the time-line reconstruction of violent criminal events.

## 1. Introduction

In the forensic field, the temporal reconstruction of the events represents a key point for the correct interpretation of the crime scene as it allows the revealing of the dynamics of the criminal event. In homicide cases, the determination of the post-mortem interval (PMI) through the classic triad (*livor*, *frigor*, *rigor mortis*) is the most used factor for timeline determination in the early period (up to 72 h). However, there is objective evidence present in all violent crime cases that can give important temporal information even in the longer term, and that is the blood. Indeed, blood can be the primary body fluid at many crime scenes and the crucial information that a bloodstain can provide is extensive and of particular importance with the emerging analytical and bio-analytical approaches.

Over the years, several studies have been carried out to identify an effective methodology that would allow the dating of a bloodstain [1,2]. All the methods proposed so far confirm that the physical and chemical properties of the bloodstain change over time, and in effect most attempts to estimate the age of a bloodstain have focused on the spectral changes of hemoglobin [3], on changes in enzymatic activity [4], on erythrocytes’ elasticity changes [5], or, more generally, on the degradation levels that occur over time for all macromolecules (DNA, RNA and proteins) [6]. A biochemical approach was also attempted through the analysis of circadian biomarkers [7]. However, none of these approaches has shown the reliability required to be used in forensic practice. In recent years, several novel techniques have been explored to determine the age of bloodstains with predictive power from a few hours up to four months, including raman spectroscopy [8,9], ATR-FTIR spectroscopy [10,11], fluorescence lifetime imaging [12,13], vibrational spectroscopy [14], infrared spectroscopy [15], and colorimetric analysis [16,17].

Despite all attempts, no technique is currently used to estimate the time since deposition (TSD) of a bloodstain in forensic practice, basically because each of these techniques is characterized by limitations in predictive power, poor analytical sensitivity and inadequate resolution between the age of the spots, and also for their complexity and costs. In addition, transferring the methodologies from laboratory conditions to real cases implies variable environmental conditions that will contribute to the complexity and difficulty of estimating the age of the bloodstain.

In the present research, a colorimetric methodology was used for age estimation of bloodstains using color change over time caused by the oxidation of blood. We have chosen to use a colorimeter that guarantees both ease of use and high precision as the detected color is converted into quantitative coordinates according to international standards. This methodology, currently used in various fields (from industrial production to cultural heritage), allows for the definition of the color of a sample in an objective, univocal and reproducible way, allowing a fine tuning of the changes over time, even for long periods. Our pilot study represents a starting point to evaluate the feasibility and reliability of the colorimetric methodology applied to forensic studies for future research.

## 2. Results

Color change over time in eight bloodstains was measured using two different color spaces, namely CIELAB and CIELCh, thus quantifying each color shade according to CIELAB colorimetric coordinates L, a, and b, as well as CIELCh coordinates C and h (details in methods). Measurements were taken once every hour for the first 24 h (except from the thirteenth to the twentieth hour) and then once a day for the remaining 60 days (details in methods).

**Figure 1:**
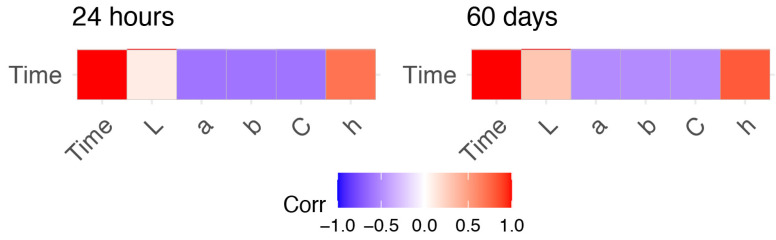
Heatmap of the level of correlation of the five analysed colorimetric coordinates with time since deposition. Positive and negative correlations are depicted in red and blue color schemes, respectively. Higher correlations are shown by dark tonalities, whereas weak correlations are represented by light colors.

First, we quantified the Pearson correlation coefficient between each pair of colorimetric coordinates, as well as between colorimetric coordinates and time (Figure 1).

For both observational periods, the h* parameter of the CIELCh space color correlated highly with TSD with correlation values of 0.67 (within 24 h) and 0.83 (within 60 days). The a*, b*, and C* parameters were negatively correlated with TSD with correlation values of approximately −0.60 (within 24 h) and −0.50 (within 60 days).In contrast, the L* parameter hadno significant correlation with time within the 24 h (r = 0.127, *p*-value = 0.140), and a week correlation (r = 0.293, *p*-value = 6.41 × 10^−9^) within 60 days. Consequently, all color coordinates but L* were used as input for building predictive models able to estimate bloodstain age.

### Bloodstain Age Estimation Models

We aimed at developing statistical/machine learning models able to estimate the TSD of a bloodstain on the basis of its color. To this end, we used and contrasted against each other five different statistical/machine learning approaches. Each approach was applied on data from four randomly chosen bloodstains, namely the training set, for deriving predictive models whose predictive capabilities were then assessed on the data from the remaining four bloodstains (test set). The whole training and test process was repeated five times, each time splitting the data differently. Each split was performed so as to ensure that the distribution of bloodstain TSD was roughly the same between the two sets, as well as to ensure that data from the same bloodstain was not present in both training and test sets [18].

The first approach was a multiple linear regression model (MLR) using the colorimetric coordinates as independent variables. The second one was a multiple quadratic regression (MQR), including the single colorimetric coordinates along with their pairwise interactions and their square powers. The third approach was a support-vector machine for regression with Gaussian kernel (SVMr), while the fourth approach was a SVM with polynomial kernel (SVMp). Finally, we trained a multiple linear model using the first two principal components (PCs) derived through principal components analysis (PCA).For ensuring an unbiased assessment of this approach, the PCs loadings derived from the training set were used for computing the PCs on the test set as well. The predictive capability of each bloodstain age prediction model was evaluated through the mean absolute deviation (MAD), Pearson correlation (r) and correct classification rate (CCR).

**Figure 2:**
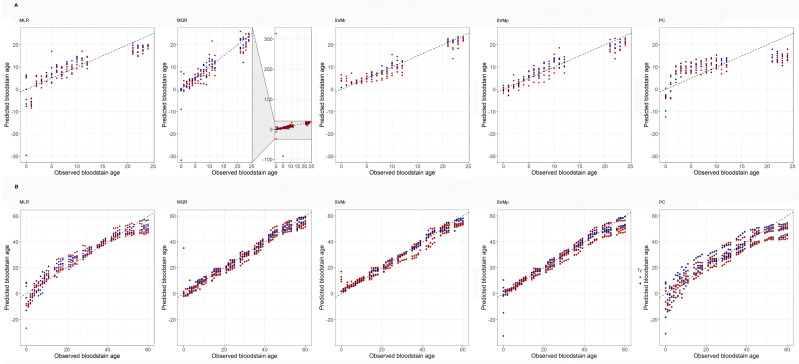
Scatterplots contrasting observed and predicted bloodstain ages. Each dot corresponds to a single prediction, either on the training set (blue dots) or on the test set (red dots). (**A**) Predictions generated on the short-term period (0 to 24 h). Scatterplots correspond to five ML approaches, which are, from left to right: Multiple Linear Regression (MLR); Multiple Quadratic Regression (MQR); Support Vector Machine with radial kernel (SVMr); Support Vector Machine with polynomial kernel (SVMp), and Principal Components (PC). (**B**) As in (**A**), but for the long-term period (1 to 60 days).

The results obtained within both observational periods for each tested model are shown in Table 1. Within the 24 h observational period, the model giving the best age prediction accuracy on both the training and test set was the SVMr, with an error less than three hours in over 70% of test samples, a MAD of about two hours and a correlation coefficient of 0.93. The poor performance of the MQR model on the test set was mainly due to the influence of outliers with abnormally large errors (Figure 2). Once these outliers were removed, the MQR model validation results were much closer to the ones of the other models (r = 0.680, MAD = 3.42 and CCR = 66.0%). The performances of other models were not significantly affected by outliers.

The results obtained within the 60 day observational period showed that the model giving the best age prediction accuracy on both the training and the test set was once again the SVMr with an error less than three days in over 60% of samples, a MAD of about three days and a correlation coefficient of 0.98.

## 3. Discussion

In forensic science, age determination of bloodstains can be decisive in reconstructing homicide cases and, more generally, in all violent crimes. To estimate TSD, changes in the color of blood outside the body have been and are often used in an approximate manner, as over time bloodstains transit from bright red to dark brown. Indeed, as the blood leaks from the vessels, hemoglobin saturates with ambient oxygen and auto-oxidizes to met-Hb and then denatures to hemichrome. The kinetics of these processes have been extensively analyzed, revealing that the oxidation of oxyhemoglobin in bloodstains follows a biphasic decay with a positive correlation of both temperature and humidity [3]. However, to obtain scientifically proven data that is useful in forensic practice, an exact estimation of timeline color variation is needed. In this work, we have employed a methodology already widely used in different research fields, the colorimetric method, that it has allowed us to develop bloodstain age prediction models in a simple, objective and also non-destructive way. It is suitable that the techniques applied on a crime scene are as non-invasive as possible both to avoid contamination and to allow for any new analyses.

The results obtained within the 24 h observational period showed that TSD predictions strongly depend on the adopted model. Using the best-performing SVMr model, the technique predicted the ages of test samples with a MAD of about two hours, a correlation coefficient of 0.93, and an error less than three hours in over 70% of samples. Using the MQR model, the technique predicted the ages of test samples with a MAD of about nine hours, a correlation coefficient of 0.06, and an error less than three hours in over 60% of samples.

Although a comparison with previous methods is made difficult by the absence of clear quantitative accuracy, it is evident that our simple and easy to use approach allows for the obtaining of a notable strictness both in the short and in the longer term periods. Using hyperspectral image analysis, Li and coworkers [19] reported an accuracy in TSD dating of 84% (CCR 83.9%) only in the first seven days with an increasing error, especially after 14 days. Kumar and coworkers [15] presented models with accuracy similar to our methodology (error in estimated date ~3–4 ± 1 days) using a much more complex method than ours (ATR-FTIR spectroscopy coupled with chemometric methods) and not practicable directly at the crime scene. Indeed, the use of a colorimeter would allow direct measurements to be made without even moving the object from its original position, avoiding any type of interference. Other complex and expensive experimental methodologies (ATR-FTIR [10], Raman Spectroscopy [20,21]) did not show particular accuracy in age estimation either in the short or the long-term.

The results obtained within the 24 h observational period showed that TSD predictions strongly depend on the adopted model. In fact, using the SVMr, the technique predicted the ages of test samples with a MAD of approximately two hours, a correlation coefficient of 0.94, and an error less than three hours in over 70% of samples (CCR =70.6%). Using the MQR model, the technique predicted the ages of test samples with a MAD of about nine hours, a correlation coefficient of 0.06, and an error less than three hours in over 60% of samples.

In recent years, a Korean group used a colorimetric method to analyze the aging of bloodstains in the first three days after deposition on a variety of supports [16,17] by an imaging and analysis integrated device. Their method involved acquiring images of bloodstains using a smartphone, but this implied that factors such as the type/quality of the lighting source and image capture distance can influence the colorimetric analysis. The colorimeter we used instead resets these external influences as the measurement is carried out through a system integrated into the instrument (a closed chamber with constant lighting and distance) that allows for standard measurements. Since the colorimetric analysis has been applied to a surface that is not perfectly smooth but has roughness due to the texture of the fabric, the light beam, even if projected on the same point, can give reflectance results that are not always the same, so, in order to obtain a robust and reliable evaluation which can be used for subsequent comparisons, we performed five measurements for each point. The colorimetric calculation was performed on the average spectrum of each point and the standard deviation was calculated for each point. We have chosen to use the fabric as a support because at crime scenes bloodstains can often be found on textile supports (clothes, sheets, furniture upholstery, etc.) but above all because when the bloodstains are completely absorbed the dehydration and aging process is uniform. Indeed, when the blood is deposited on not completely permeable materials, the aging process and therefore the chromatic variation is greatly influenced by different dehydration processes with the formation of inhomogeneous areas.

The high capability and reliability demonstrated by our method has enabled us to perform both short- and long-term measurements. The literature data currently available on the estimate of the age of blood stains by exploiting color variations are limited from the first hours [16,17] up to a maximum of eight days [1] and 30 days [19], while in our work we have documented detectable color variations up to 60 days.

Clearly, bloodstains left on different fabrics (texture, composition, color of the fabric) as well as the effects of environmental conditions (temperature, humidity, sunlight) will require additional analysis for accurate TSD predictions. Moreover, further validation studies involving an evaluation of actual casework samples would be required.

In conclusion, in this work we verified the ability and reliability of colorimetric analysis in dating the age of a bloodstain for forensic purposes. Although preliminary, our results show that this methodology can be considered fully adequate and up to the requirements of forensic medicine both in terms of reliability and practicability. To increase the general impact of our data, it would be necessary to increase the number of samples used and also to evaluate different experimental conditions (temperature, humidity, etc.). However, our work underscores the possibility of providing investigators of violent crimes with a non-destructive, simple, and objective method for estimating the age of bloodstains at the crime scene.

## 4. Materials and Methods

### 4.1. Preparation of the Blood Samples

**Figure 3:**
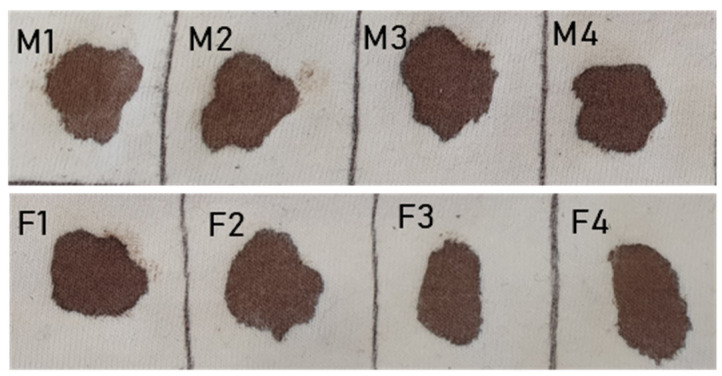
Set of bloodstains of the 8 subjects (males M1–4 and females F1–4) at the sixtieth day of aging.

For preparation of bloodstains, we used untreated whole blood from healthy volunteers (four males and four females) aged between 25 and 35 years. Blood samples were obtained by capillary sampling with blood lancets and bloodstains were made immediately by dropping a few drops from the fingertip on the target substrate (100% white cotton fabric). We created 40 bloodstains (five stains from each subject) with an average diameter of 17.0 mm and a volume of 0.5 mL (Figure 3). To simulate an indoor crime scene, we placed the blood samples on tissue in an air-conditioned room at 25° C during the day (from 8 a.m. to 8 p.m.) with natural lighting but not in direct sunlight. Measurements were carried out over a period of two months (May and June 2020).

### 4.2. Colorimetric Analysis of the Blood Samples

Bloodstain colorimetric analysis for age estimation was detected by using a spectrophotometer (3NH, NS800, SHENZHEN TreeNH TECHNOLOGY CO., LTD, SHENZHEN, P.R. China) with a spot diameter of 8 mm. The lighting system, color evaluation and calculation formulas were set upstream of the measurement and the results were obtained directly as CIELAB/CIELCh parameters. During the first 24 h, we performed a measurement every hour (except from the thirteenth to the twentieth hour) while, thereafter, we measured the bloodstains once a day, around the same time the stains were originally laid, for the next 60 days. Each bloodstain was scanned five times and only the average of the five measurements was used for the following analyses. After the bloodstains scan, the spectrophotometer system returns the color intensity in two different color spaces: the CIELAB and CIELCh. In the CIELAB color space, the L* coordinate represents the lightness dimension and ranges from 0 to 100, with 0 being black and 100 being white. The red/green colors are represented along the a* coordinate, with green at negative a* values and red at positive a* values. The yellow/blue colors are represented along the b* coordinate, with blue at negative b* values and yellow at positive b* values. The CIELCh space is a color space based on CIELAB, which uses the polar coordinates C* (chroma, relative saturation) and h* (hue angle, angle of the hue in the CIELAB color wheel) instead of the Cartesian coordinates a* and b*. The conversion of a* and b* to C* and h* is based on the following formula:

$$C^{*}=\sqrt{a^{*2}+b^{*2}};\;h=\arctan\left(\frac{b^{*}}{a^{*}}\right)$$

All five parameters of the CIELAB and CIELCh color spaces (L*, a*, b*, C* and h*) were used to predict the TSD of the bloodstains.

### 4.3. Statistical Analyses

Two different time horizons were adopted to investigate the change in color intensity of a bloodstain over time: the first one was based on the data obtained in the first 24 h of observation, and the second one was based on the data obtained in the first 60 days. For each time horizon, machine learning models were used for predicting bloodstain age on the basis of the five parameters (L*, a*, b*, C* and h*) of the CIELAB and CIELCh colorimetric models.

First, a training set was created by randomly selecting four bloodstains. Data from the remaining bloodstains were included in the corresponding test set. Five different machine learning approaches were then used for deriving predictive models on the training set, while the predictive capabilities of these models were assessed on the test set. The whole process was repeated five times with different data splits, so as to gauge the associated variance. Notably, during each split each bloodstain was assigned either to the training or to the test set. In such a way we ensured that (a) both sets cover equal time periods, and (b) data from the same bloodstain was not used at the same time for training and validation, so as to guarantee an unbiased estimation of predictive performance [18].

The first machine learning approach was based on a multiple linear regression model (MLR) using the single colorimetric coordinates as independent variables. The second one was a multiple quadratic regression (MQR), including the single colorimetric coordinates along with their pairwise interactions and their square powers. The third and fourth approaches were based on support vector machines for regression (SVM): the first one exploited a Gaussian kernel (SVMr), the second one a polynomial kernel (SVMp). Both these SVM non-linear machine learning algorithms were able to capture complex patterns and interactions within data [22]. Finally, we trained a multiple linear model using the first two principal components (PCs) derived through principal component analysis (PCA) of the colorimetric coordinates [23]. For ensuring an unbiased assessment of this approach, the PCs loadings derived from the training set were used for computing the PCs on the test set as well. The predictive capability of each bloodstain age prediction model was evaluated through the mean absolute deviation (MAD), Pearson correlation (r) and correct classification rate (CCR). MAD provides an immediately comprehensible quantification of the prediction error in terms of how many hours/days each prediction is, on average, off. The Pearson correlation indicates how well predictions and observed values vary together. With respect to the CCR, the obtained results were evaluated as either correct (if the predicted age was concordant with the TSD ±three hours in the short period or ±three days in the long period), or incorrect (if these values differed by more than three units of measurement).The final performances are reported as the average and standard deviation over the five different splits. Outliers were identified as measurements whose standardized residuals were larger than three units (in absolute value).

All statistical analyses and graphical representations were performed using R v 4.04 (https://www.r-project.org/, accessed on 24 May 2021), using a custom script.

## Figures and Tables

**Table 1:** Predictive performances of the bloodstain age estimation models. For each model the correlation coefficient r, the mean absolute deviance (MAD) and the correct classification rate (CCR) metrics are reported, both for the 24 h and 60 days prediction tasks. MLR: Multiple Linear Regression; MQR: Multiple Quadratic Regression; SVMr: Support Vector Machine with radial kernel; SVMp: Support Vector Machine with polynomial kernel; PCs: Principal Components.

	Period											
	24 h						60 days					
	Training			Test			Training			Test		
Prediction Models	r	MAD	CCR	r	MAD	CCR	r	MAD	CCR	r	MAD	CCR
MLR	0.902 (0.009)	2.70(0.25)	59.1 (6.69)	0.743 (0.041)	3.82 (0.32)	53.8 (4.4)	0.964 (0.003)	3.80 (0.19)	49.5 (2.8)	0.957 (0.005)	3.92 (0.35)	52.6 (6.0)
MQR	0.967 (0.007)	1.54(0.16)	87.6 (2.23)	0.281 (0.193)	8.93 (5.79)	64.7 (6.66)	0.993 (0.001)	1.72 (0.17)	82.8 (3.9)	0.820 (0.135)	3.48 (0.65)	64.7 (4.1)
SVMr	0.982 (0.006)	1.12(0.18)	94.1 (2.75)	0.933 (0.012)	2.29 (0.27)	71.2 (5.6)	0.995 (0.001)	1.56 (0.10)	91.5 (2.8)	0.982 (0.003)	3.05 (0.18)	60.6 (3.7)
SVMp	0.961 (0.006)	1.69(0.19)	80.0 (2.46)	0.942 (0.013)	2.32 (0.28)	69.7 (6.5)	0.988 (0.003)	2.24 (0.36)	74.3 (9.0)	0.974 (0.002)	3.17 (0.09)	58.7 (3.6)
PCs	0.682 (0.010)	4.85 (0.11)	29.7 (2.83)	0.687 (0.005)	4.80 (0.09)	32.9 (4.0)	0.944 (0.008)	4.79 (0.42)	41.1 (5.7)	0.941 (0.008)	4.99 (0.25)	43.2 (4.7)

Note: performances are reported as the average and standard deviation over the five different random splits.

## Data Availability

All data, models, and code that support the findings of this study are available as a Supplementary Material.

## Supplementary Materials

The information are available online.
