# Supplementary figures and images for: Short and Long Time Bloodstains Age Determination by Colorimetric Analysis: A Pilot Study
Source: Molecules. 2021 Oct 16;26(20):6272. doi: 10.3390/molecules26206272 (PMC8540217; doi:10.3390/molecules26206272)

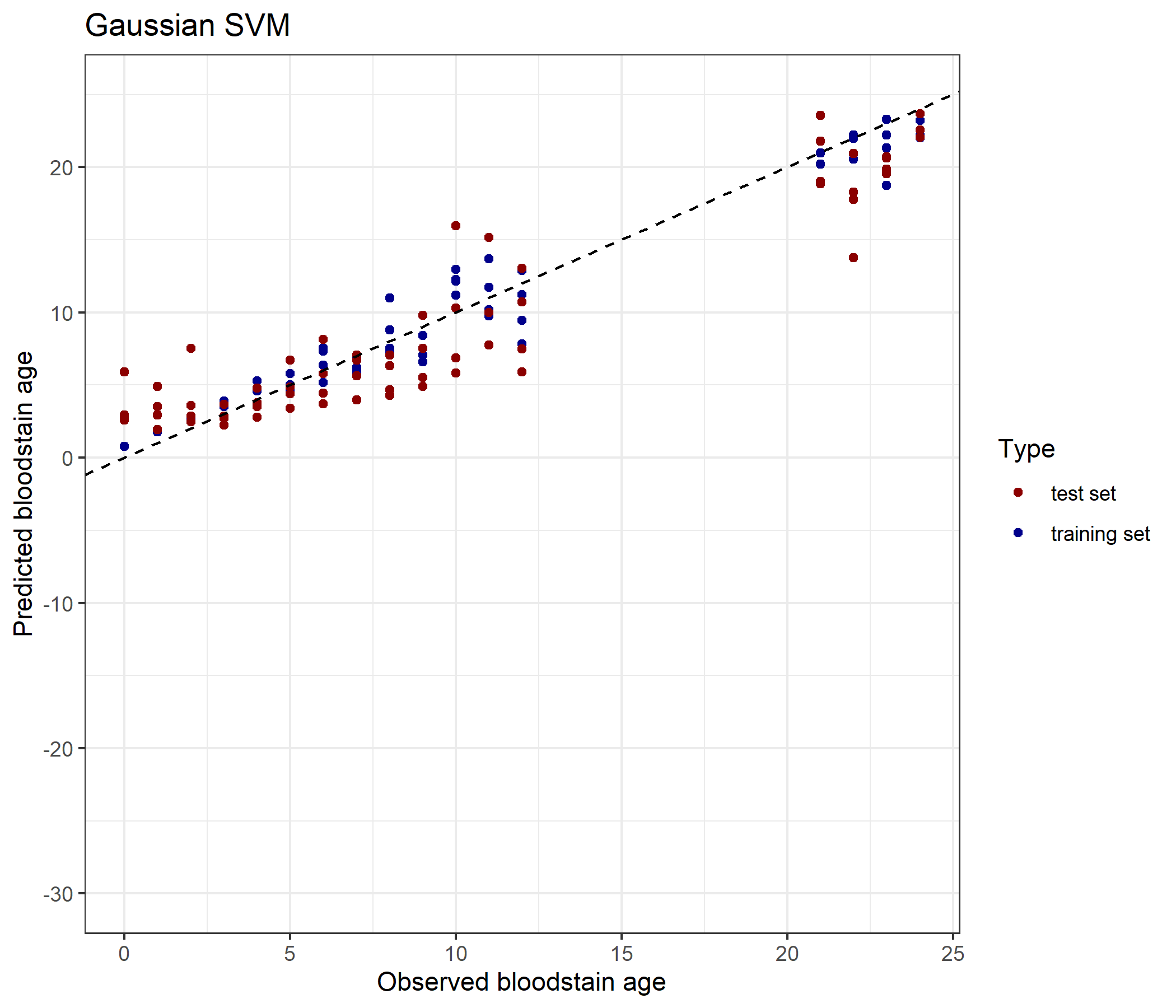

Supplement: Supplementary file 1 [file molecules-26-06272-s001.zip › molecules-1408359-supplementary/to_release/res_24h/gaussian_svm_repetition_5.png]

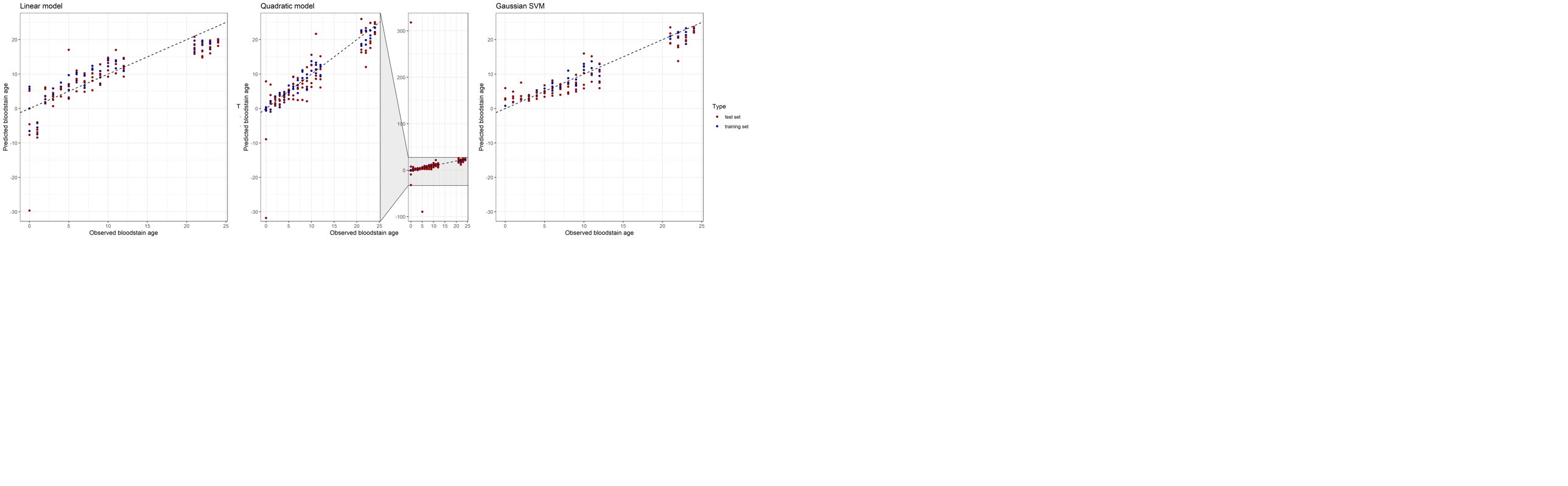

Supplement: Supplementary file 1 [file molecules-26-06272-s001.zip › molecules-1408359-supplementary/to_release/res_24h/linear_model_repetition_5.png]

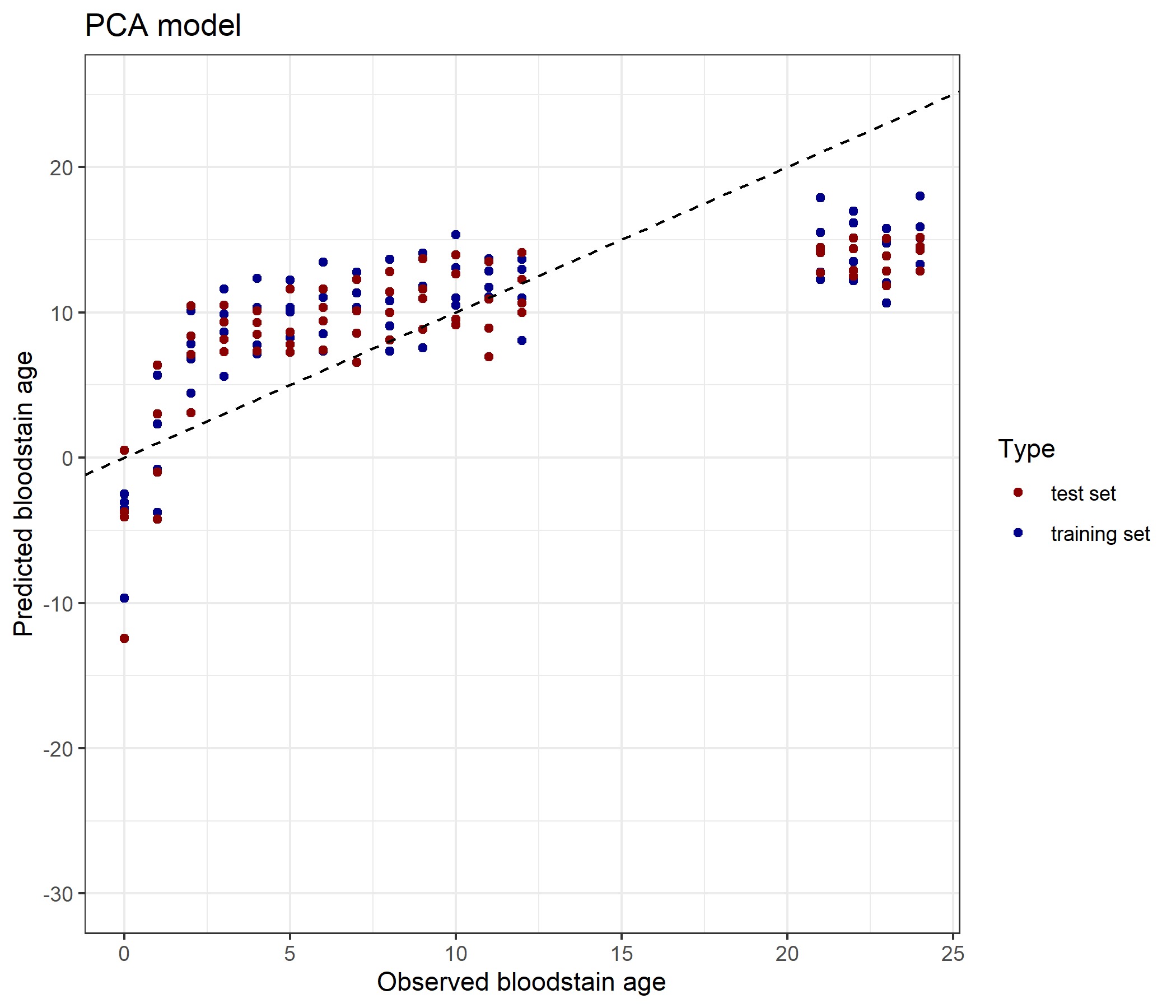

Supplement: Supplementary file 1 [file molecules-26-06272-s001.zip › molecules-1408359-supplementary/to_release/res_24h/pca_model_repetition_5.png]

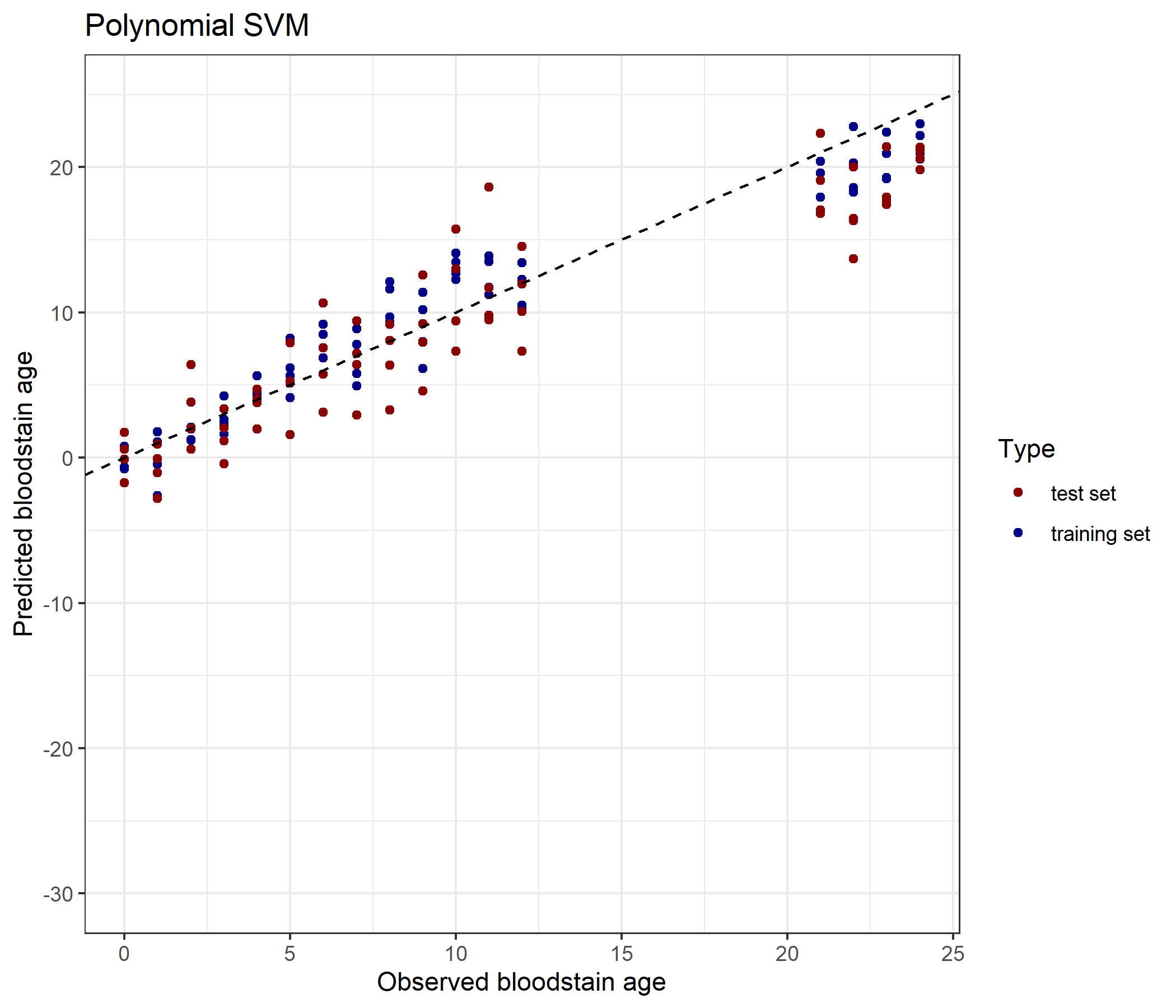

Supplement: Supplementary file 1 [file molecules-26-06272-s001.zip › molecules-1408359-supplementary/to_release/res_24h/polynomial_svm_repetition_5.png]

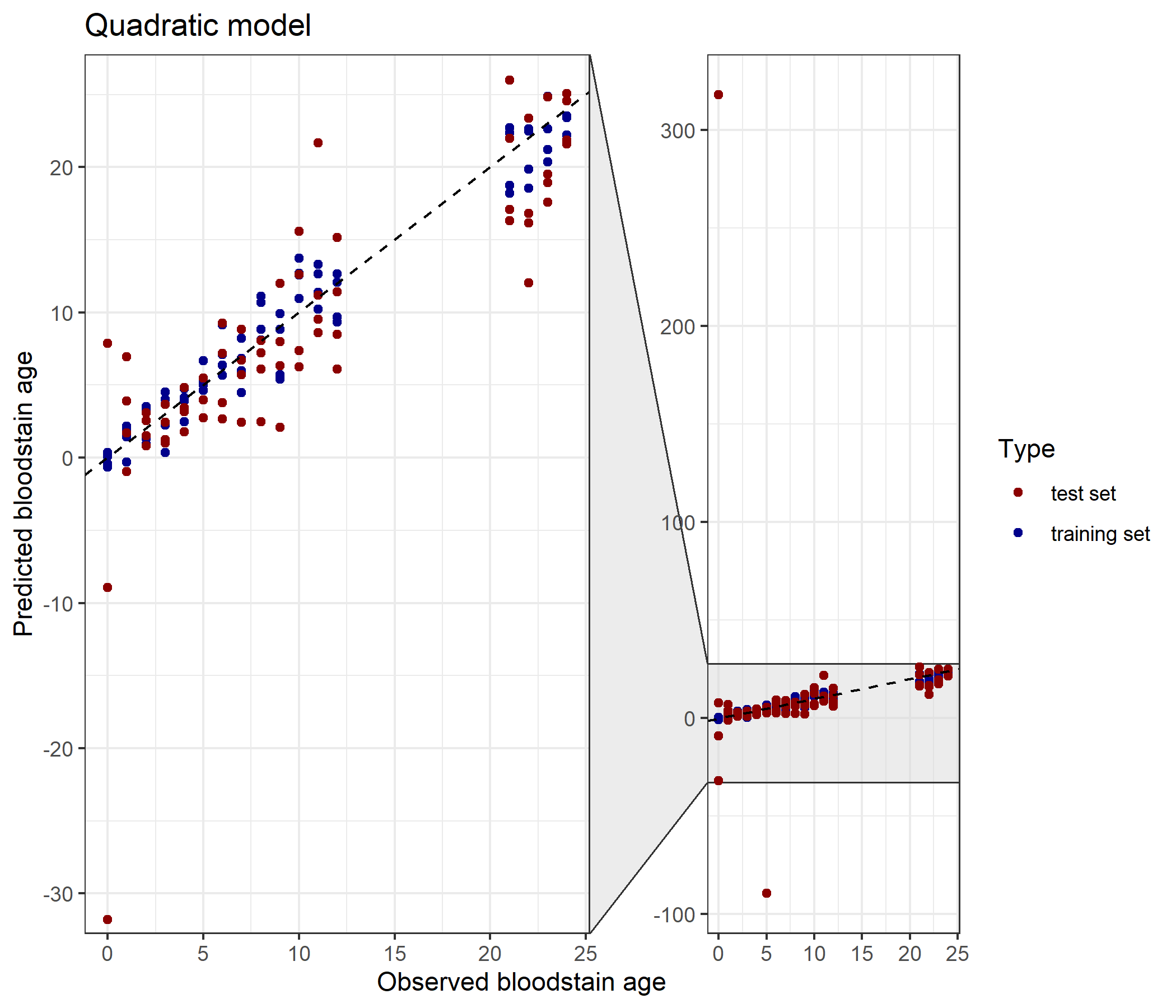

Supplement: Supplementary file 1 [file molecules-26-06272-s001.zip › molecules-1408359-supplementary/to_release/res_24h/quadratic_model_repetition_5.png]

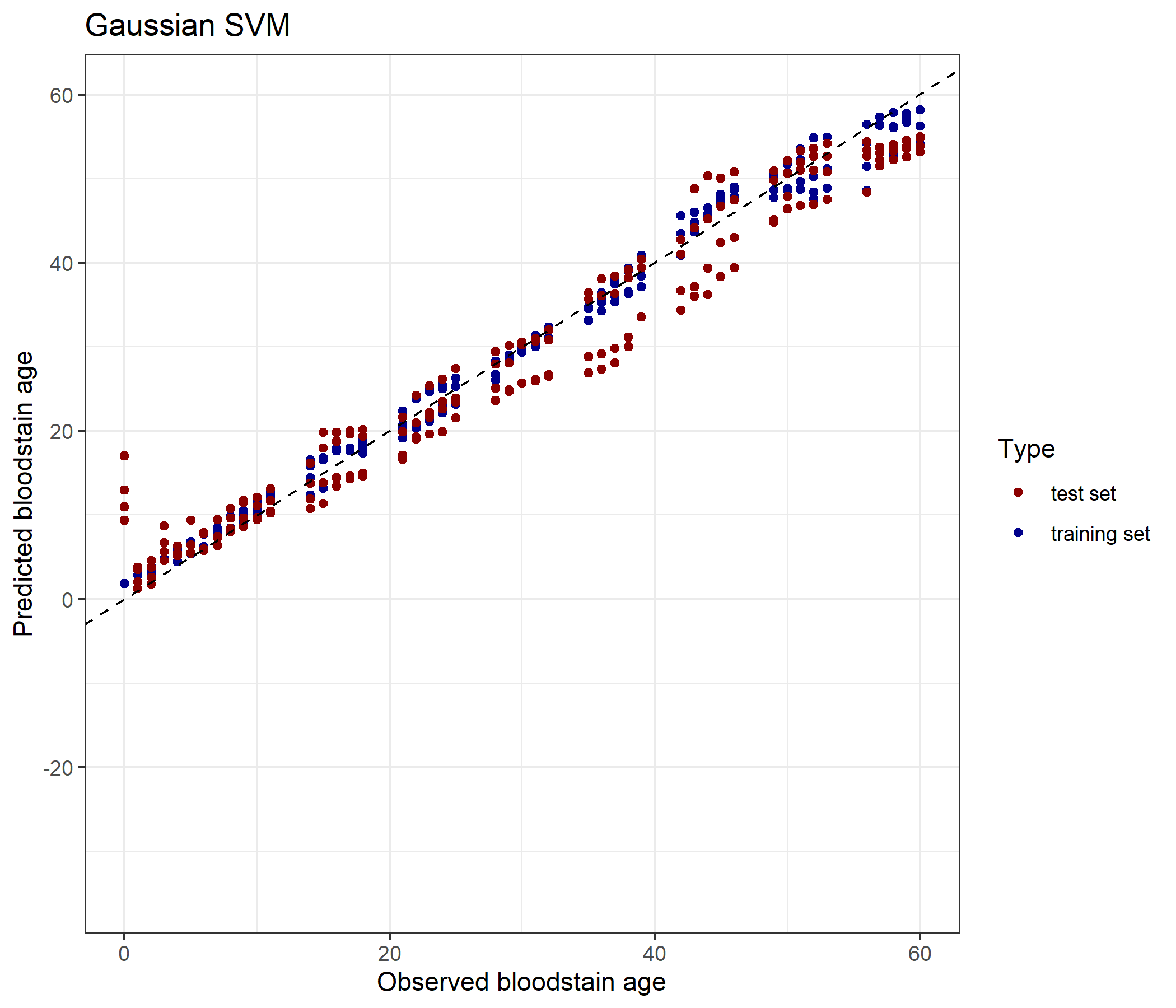

Supplement: Supplementary file 1 [file molecules-26-06272-s001.zip › molecules-1408359-supplementary/to_release/res_60d/gaussian_svm_repetition_5.png]

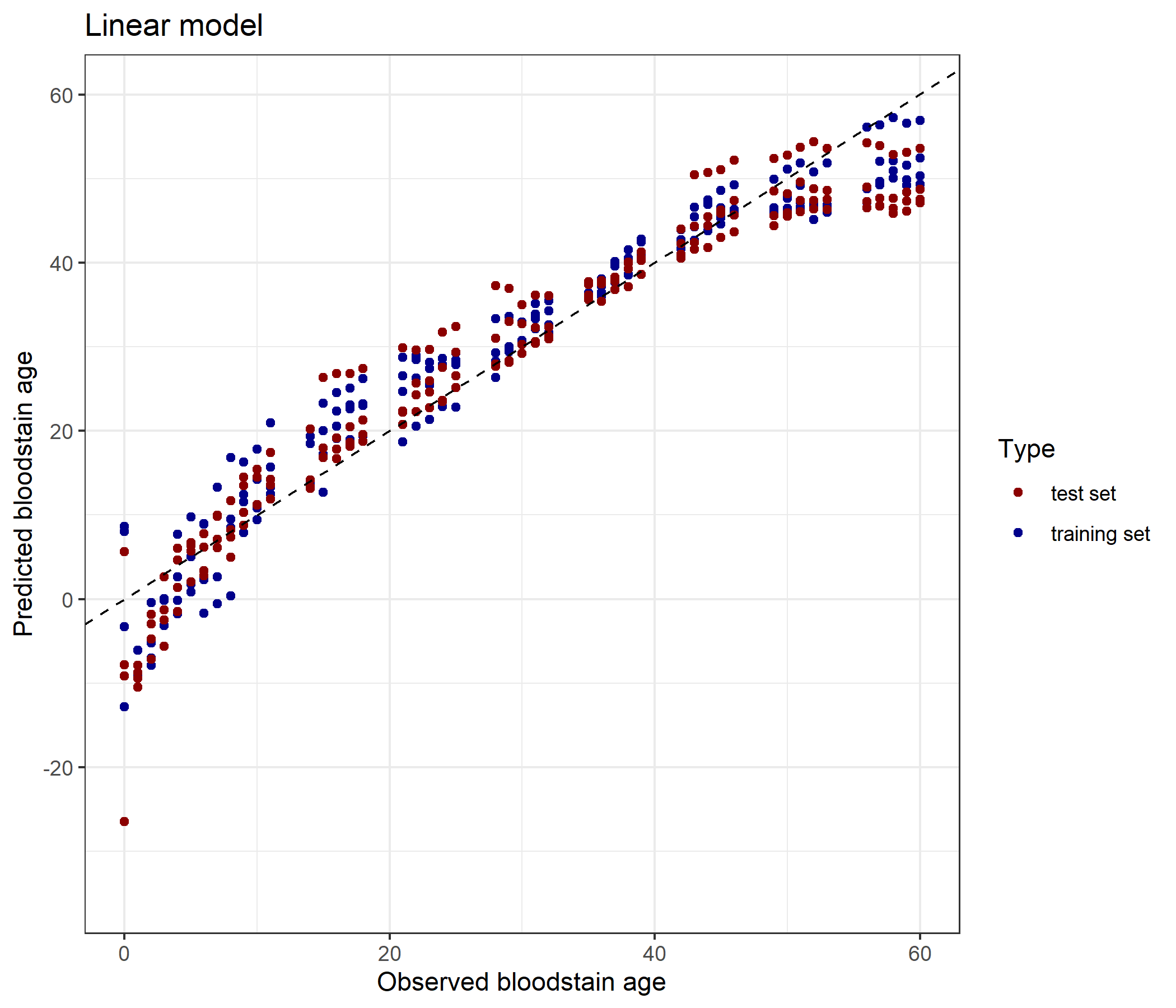

Supplement: Supplementary file 1 [file molecules-26-06272-s001.zip › molecules-1408359-supplementary/to_release/res_60d/linear_model_repetition_5.png]

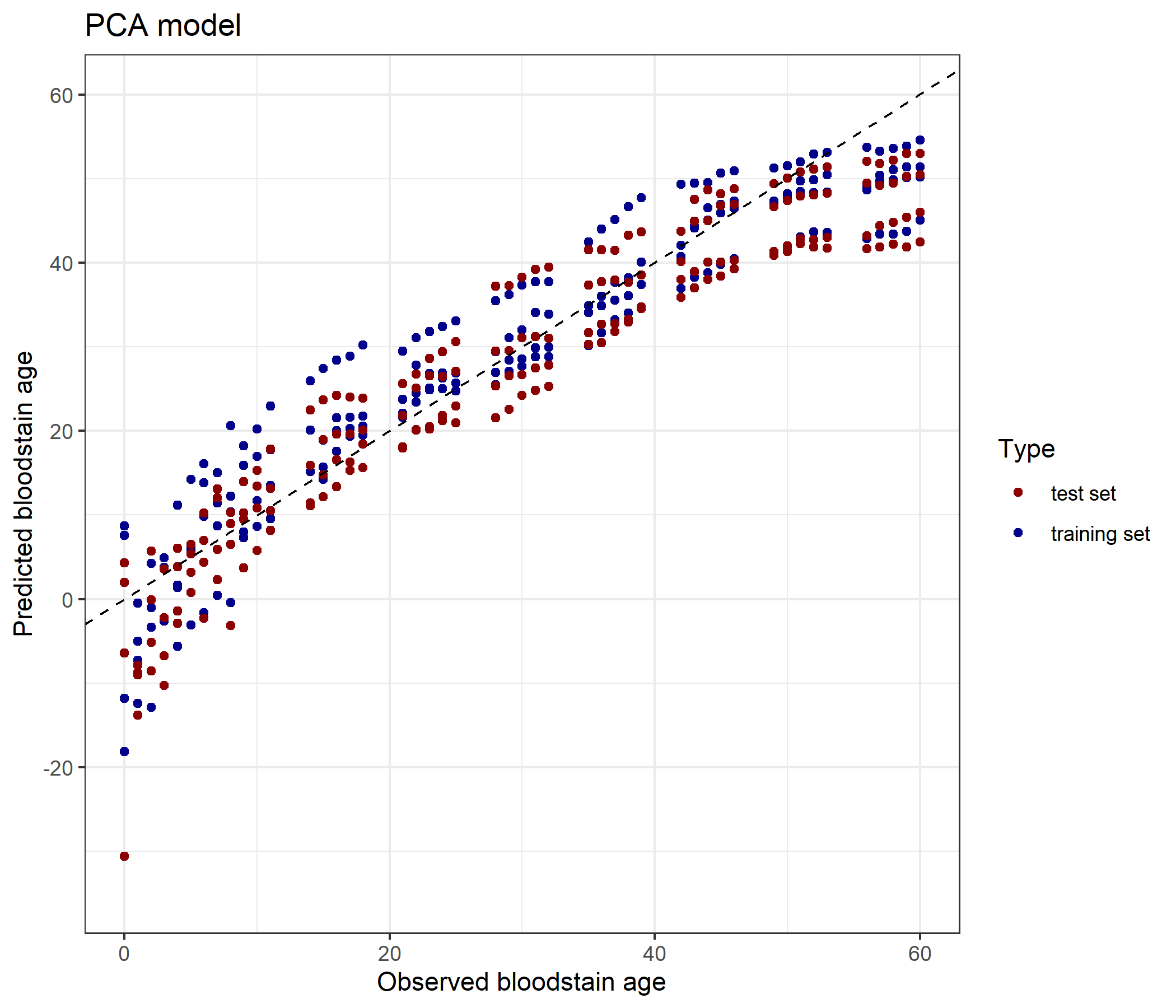

Supplement: Supplementary file 1 [file molecules-26-06272-s001.zip › molecules-1408359-supplementary/to_release/res_60d/pca_model_repetition_5.png]

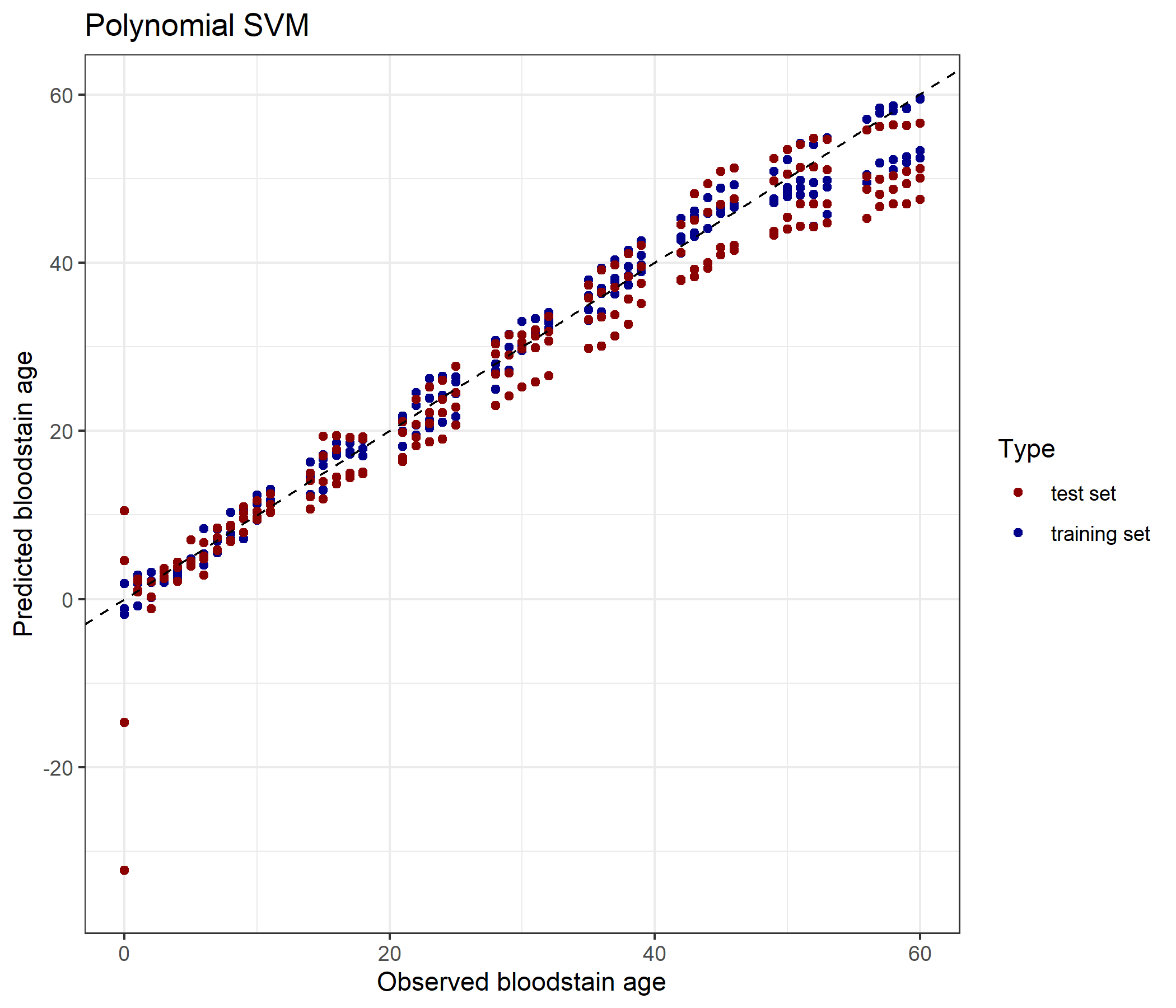

Supplement: Supplementary file 1 [file molecules-26-06272-s001.zip › molecules-1408359-supplementary/to_release/res_60d/polynomial_svm_repetition_5.png]

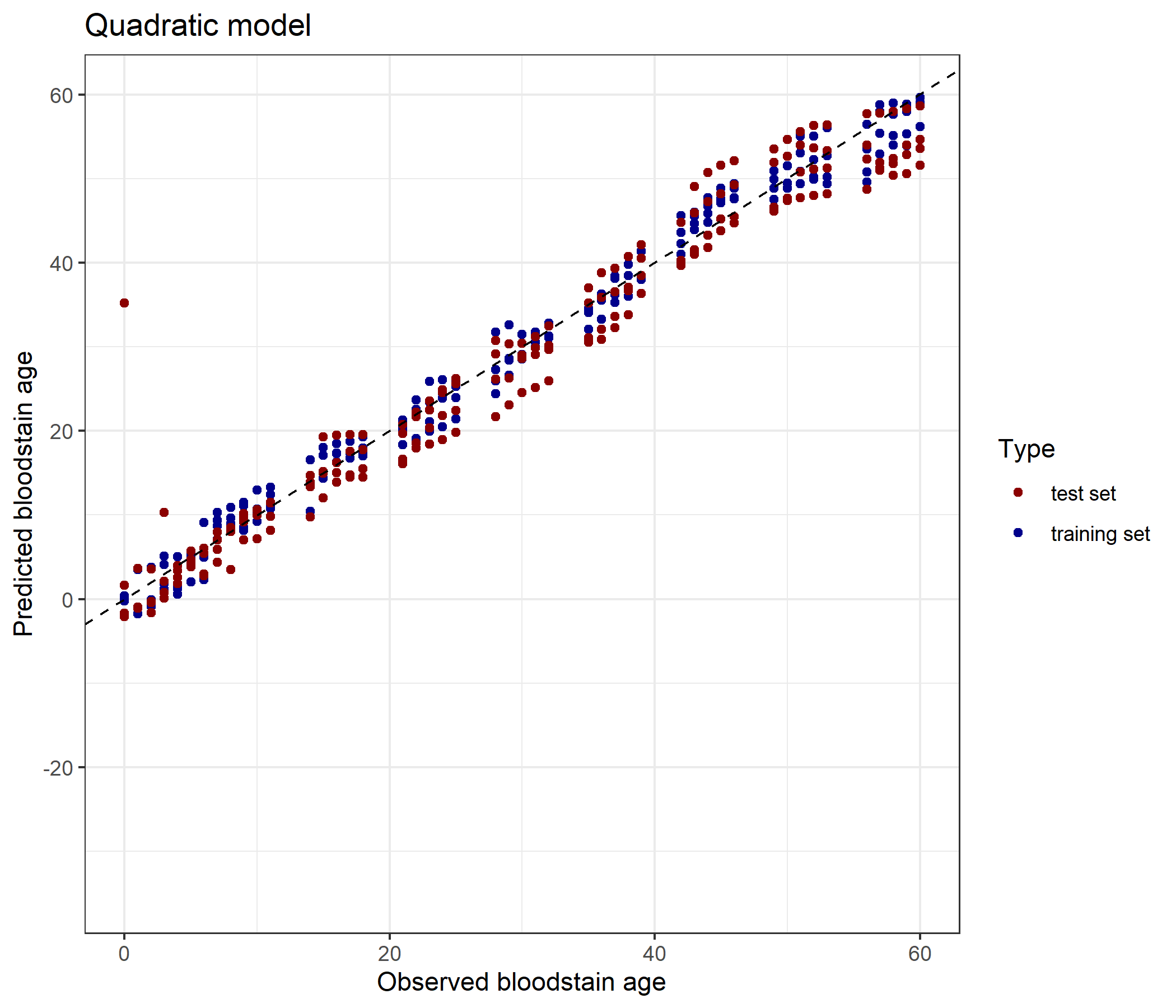

Supplement: Supplementary file 1 [file molecules-26-06272-s001.zip › molecules-1408359-supplementary/to_release/res_60d/quadratic_model_repetition_5.png]
